# Prognostic indices in diffuse large B-cell lymphoma: a population-based comparison and validation study of multiple models

**DOI:** 10.1038/s41408-023-00930-7

**Published:** 2023-10-13

**Authors:** Jelena Jelicic, Karen Juul-Jensen, Zoran Bukumiric, Michael Roost Clausen, Ahmed Ludvigsen Al-Mashhadi, Robert Schou Pedersen, Christian Bjørn Poulsen, Peter Brown, Tarec Christoffer El-Galaly, Thomas Stauffer Larsen

**Affiliations:** 1 Department of Hematology, Vejle Hospital, Sygehus Lillebaelt, Vejle, Denmark; 2 Department of Hematology, Odense University Hospital, Odense, Denmark; 3 Institute for Medical Statistics and Informatics, University of Belgrade, Faculty of Medicine, Belgrade, Serbia; 4 Department of Hematology, Aarhus University Hospital, Aarhus, Denmark; 5 Department of Hematology, Aalborg University Hospital, Aalborg, Denmark; 6 Department of Hematology, Regional Hospital Gødstrup, Herning, Denmark; 7 Department of Hematology, Zealand University Hospital, Roskilde, Denmark; 8 Department of Hematology, Copenhagen University Hospital, Rigshospitalet, Denmark; 9 Department of Clinical Research, University of Southern Denmark, Odense, Denmark

**Keywords:** Lymphoma, Cancer models

## Abstract

Currently, the International Prognostic Index (IPI) is the most used and reported model for prognostication in patients with newly diagnosed diffuse large B-cell lymphoma (DLBCL). IPI-like variations have been proposed, but only a few have been validated in different populations (e.g., revised IPI (R-IPI), National Comprehensive Cancer Network IPI (NCCN-IPI)). We aimed to validate and compare different IPI-like variations to identify the model with the highest predictive accuracy for survival in newly diagnosed DLBCL patients. We included 5126 DLBCL patients treated with immunochemotherapy with available data required by 13 different prognostic models. All models could predict survival, but NCCN-IPI consistently provided high levels of accuracy. Moreover, we found similar 5-year overall survivals in the high-risk group (33.4%) compared to the original validation study of NCCN-IPI. Additionally, only one model incorporating albumin performed similarly well but did not outperform NCCN-IPI regarding discrimination (c-index 0.693). Poor fit, discrimination, and calibration were observed in models with only three risk groups and without age as a risk factor. In this extensive retrospective registry-based study comparing 13 prognostic models, we suggest that NCCN-IPI should be reported as the reference model along with IPI in newly diagnosed DLBCL patients until more accurate validated prognostic models for DLBCL become available.

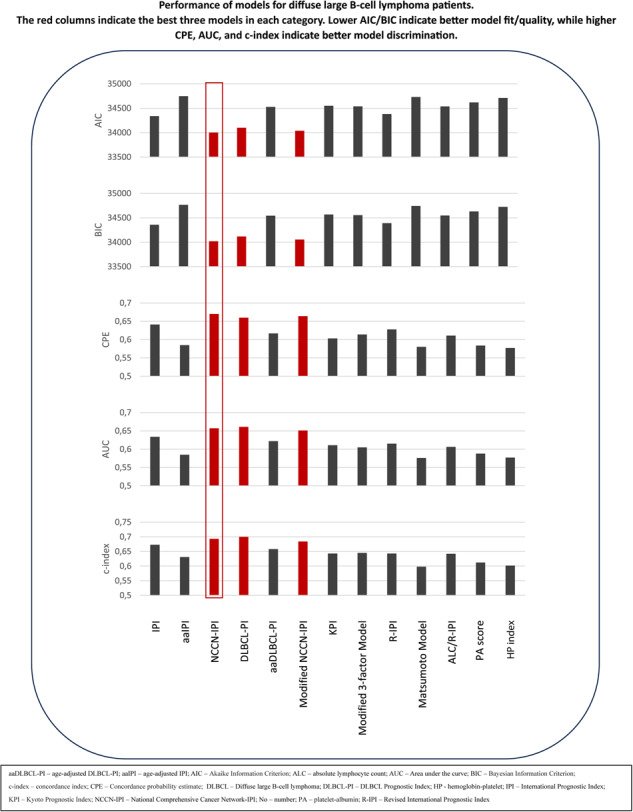

## Introduction

Diffuse large B-cell lymphoma (DLBCL) represents the most common type of non-Hodgkin lymphoma with significant heterogeneity in survival [1]. Numerous clinical prognostic models have been developed to stratify patients according to risk [2]. The International Prognostic Index (IPI) was developed to predict survival in patients with aggressive lymphoma treated with doxorubicin [3]. Although the model was published in 1993, it remains the most widely adopted prognostic tool in newly diagnosed DLBCL and the primary tool for selection and risk stratification in today’s clinical trials [4, 5].

In the original study developing the IPI, patients were stratified into four risk groups based on five variables (age, Ann Arbor stage, Eastern Oncology Cooperative Group performance status [ECOG PS], number of extranodal sites, and lactate dehydrogenase [LDH]). The 5-year overall survival (OS) ranged from 26% to 73% in high- and low-risk groups, respectively [3]. However, due to changes in treatment regimens over time, including the introduction of rituximab, the prognostic value of the IPI has declined [2, 6–8]. Moreover, recent treatment improvements, primarily in relapsed/refractory patients with the introduction of chimeric antigen receptor (CAR) T-cell therapy and other therapies under investigation, have increased the need for optimized models to predict poor-risk patients and thus further challenge the usefulness of the IPI [4, 9]. Although many biological factors correlate with disease outcomes, integration into accurate, validated prognostic models is lacking [4]. Establishing a model integrating patient characteristics through an empirical approach to estimate accurate probabilities of an outcome using accessible predictors and with better utilization of data and current devices is needed [10, 11].

Several studies developed IPI-like variants with superior discriminatory ability compared to the IPI in the rituximab era [6–8, 12]. The most commonly reported IPI-like models are the Revised IPI (R‐IPI), and the National Comprehensive Cancer Network IPI (NCCN‐IPI), although the attempts to incorporate genetic and molecular variables have shown promising results [1, 2, 13]. However, these models are difficult to reproduce due to the time-consuming data analysis processes and potential costs, which further limit the validation of such a model [14]. Available prognostic models for DLBCL are mainly developed from retrospective studies with a limited number of patients and frequently lack external validation [15]. Most studies aiming to establish DLBCL models focused on OS as the primary survival point and the model’s discrimination ability, which measures the actual probability that a given patient experienced the event (e.g., disease recurrence or death) [15, 16]. Optimal survival end-point in DLBCL patients has been a matter of debate due to improved outcomes and hence the low number of events, and event-free survival (EFS) at 24 months has been proposed as a valid surrogate marker for OS and relative survival [17]. Additionally, all-cause mortality data further challenge current models in DLBCL as these include deaths from all causes, but in older patients, non-lymphoma deaths should be considered [11]. Moreover, calibration, the agreement between observed outcomes and predictions, has gained more attention recently and is recommended when externally validating prediction models [18]. Several validation studies in DLBCL patients have been conducted [2, 8, 11, 19]. However, most of them are either based on a limited number of patients, compared to few prognostic models, lack external validation and calibration analysis, or in the case of large populations, patients were recruited from clinical trials not reflecting the real-world population [2, 8, 11, 19].

This study was conducted to identify the most accurate current prognostic model by validation in a real-world DLBCL population. We report the discrimination ability and calibration of 13 prognostic models and identify the current model that could serve as a reference when developing DLBCL models and potentially be used in patient selection in clinical trials.

## Methods

Patients were identified through the nationwide Danish lymphoma registry (LYFO), which contains information on baseline clinicopathologic features, treatment, and outcomes of DLBCL patients. LYFO coverage is 98%, and its database is periodically merged with the national civil registry, which contains the dates of death of all deceased inhabitants, providing accurate survival calculations [20]. The study was approved by RKKP (Regionernes Kliniske Kvalitetsudviklingsprogram) with study number 21/27006. Access to data was available to all authors.

Patients were included in the final analysis if they fulfilled the following inclusion criteria: (1) age ≥18 years; (2) newly diagnosed with DLBCL between January 2000 and June 2021; (3) treated with at least one cycle of R-CHOP (rituximab, cyclophosphamide, doxorubicin, vincristine, and prednisone) or similar; (4) all clinical/laboratory variables retrievable in order to calculate prognostic indices in all models. Patients with primary central nervous system (CNS) lymphoma were excluded, while patients with systemic disease and concomitant CNS involvement were included in the study.

### Prognostic models

Clinical prognostic models developed for DLBCL patients were evaluated if the variables for models were available in LYFO. We compared 13 models incorporating at least one clinical (e.g., age, ECOG PS, Ann Arbor stage) and/or laboratory variables (e.g., hemoglobin, LDH, albumin) identified through a previously published systematic review [6]. Those models were considered clinical models. Additionally, as test comparisons, two exclusively laboratory-based models incorporating only variables such as thrombocytes, hemoglobin, and albumin were calculated to compare whether the addition of clinical variables improves the performance of a model. Models including beta-2 microglobulin (β2M) were not included in the current analysis, as this laboratory marker was frequently lacking, and multiple imputations would not reasonably approximate the true distributional relation between unobserved data and available information [21].

### Statistical analysis

OS was defined as the time from diagnosis until death from any cause or censoring the last follow-up. Progression-free survival (PFS) was defined as the time from diagnosis to relapse/disease progression or censoring at the last follow-up. OS was calculated using the Kaplan–Meier method, and we used the Log-rank test to compare the difference between risk groups. Cox proportional hazard models obtained hazard ratios with 95% confidence intervals (CI).

As a measure of fit/model quality, Akaike Information Criterion (AIC) and Bayesian Information Criterion (BIC) were used. Lower AIC or BIC indicates a better fit [22]. The area under the receiver operating characteristic curve (AUC) was used as a standard method to assess the accuracy of the predictive distribution model [23]. According to Uno et al., the concordance index (c-index) was used to measure discrimination, representing a model’s ability to distinguish individuals with and without outcomes of interest [24, 25]. A value of 0.5 indicates no discrimination, while 1 indicates perfect discrimination [24]. Concordance probability estimate (CPE) was used as another discrimination measure, with higher values indicating better discrimination [26]. Calibration represents the agreement between predicted and actual probabilities estimated by a predictive model [16]. The calibration of models in the current study was presented with calibration curves. Models close to a 45-degree line show perfect calibration [16]. Interrater-weighted *κ* statistics along with 95% CI was used to compare agreement between the IPI, NCCN-IPI, and other four-risk models, and R-IPI and other models with three-risk groups [27].

All *p*-values were 2-sided, and *p* < 0.05 was considered statistically significant. Calculations were performed in IBM SPSS statistics (version 28.0.0.0) and R version 3.4.1 using the following packages for survival and performance calculations: CPE, ggplot2, ggsurvfit, dynpred, maxstat, rms, survC1, survival.

## Results

**Fig. 1:**
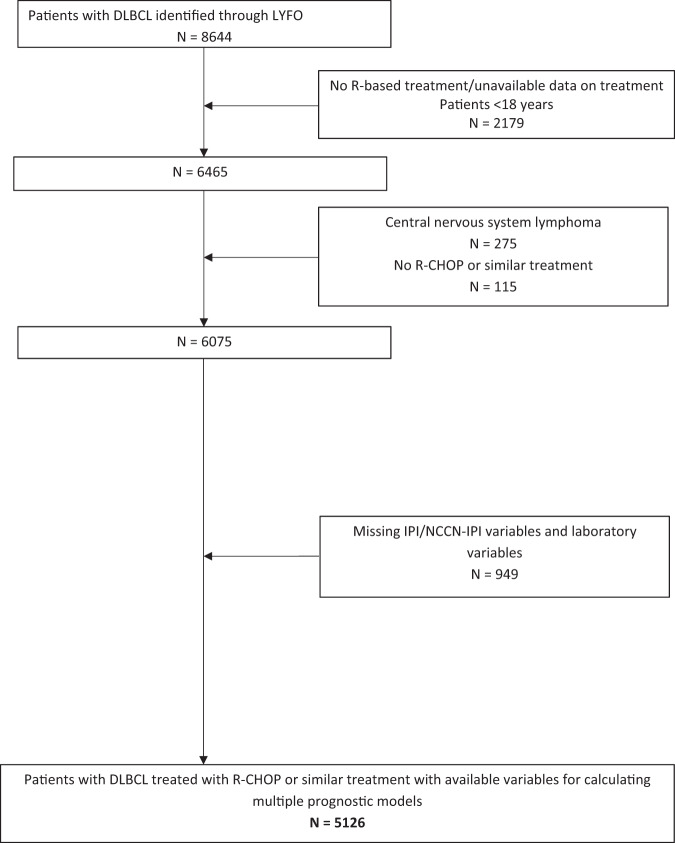
Consort diagram of the selection process for identifying patients eligible for the current study. DLBCL diffuse large B-cell lymphoma, IPI International Prognostic Index, NCCN-IPI National Comprehensive Cancer Network IPI, LYFO Danish Lymphoma Register, R rituximab, R-CHOP rituximab, cyclophosphamide, doxorubicin, vincristine, and prednisone.

Of 8644 patients registered with DLBCL in LYFO in the inclusion period, 6075 patients with DLBCL were treated with rituximab-based therapy and were considered potential candidates for the current study. Data to calculate prognostic indices of interest were available for 5126 patients who fulfilled inclusion criteria and were selected for the final analysis (Fig. 1). Data on missing variables among 6075 potential candidates is provided in Supplementary Fig. 1.

Table 1 summarizes baseline patient characteristics. The median age was 68 years (range 18–95), with 71.0% older than 60. There was a slight male predominance (57.3%) and patients with advanced stage III and IV disease (68.1%) (Table 1).

**Table 1:** Clinical characteristics of patients with diffuse large B-cell lymphoma.

Patient characteristics		*N* of patients (%) Total *N* = 5126
Age	Median (range)	68 (18-95)
	≤40	297 (5.8)
	41–60	1191 (23.2)
	61–75	2338 (45.6)
	>75	1300 (25.4)
Gender	Males	2938 (57.3)
Ann Arbor stage	I	851 (16.6)
	II	787 (15.3)
	III	916 (17.9)
	IV	2572 (50.2)
ECOG PS	≥2	857 (16.7)
B symptoms	Present	2143 (41.8)
	NA	104 (2.0)
Bulky disease	<7.5 cm	3324 (64.8)
	NA	226 (4.4)
LDH	≤ ULN	2295 (44.8)
	> 1–3xULN	2344 (45.7)
	>3x ULN	487 (9.5)
EN IPI	>1	1620 (31.6)
EN NCCN-IPI	≥1	1684 (32.9)
Hgb	Grade 2^a^	613 (12.0)
PLT	<100 × 10^9^/L	184 (3.6)
ALC	≤0.84 × 10^9^/L	1190 (23.2)
Albumin	<35 g/L	1545 (30.1)

*ALC* absolute lymphocyte count, *ECOG* *PS* Eastern Oncology Cooperative Group performance status, *EN* Extranodal, *Hgb* hemoglobin, *LDH* lactate dehydrogenase, *NA* not applicable, *N* number, *PLT* platelets, *ULN* upper limit of normal.

^a^Anemia grade 2: Hgb <10–8.0 g/dL (Common Terminology Criteria for Adverse Events—CTCAE).

### Prognostic models

We identified 13 clinical and two laboratory models from 11 studies [3, 7, 8, 12, 28–34]. All models except IPI and age-adjusted IPI (aaIPI) were developed for patients treated with rituximab-based regimens. Table 2 summarizes the variables included in each model. Tables 3 and 4 provide calculations and distributions of patients within each risk group and among our cohort according to models with four- (*n* = 8) and three-risk groups (*n* = 5) [3, 7, 8, 12, 28–34]. All studies except two included DLBCL patients from retrospective cohorts [3, 28]. Slight differences in inclusion criteria with variable follow-up under 60 months were reported across the studies. The number of patients from the original populations used to develop current models ranged from 88 to 2031 [3, 28]. The largest study population was used to develop IPI, followed by DLBCL Prognostic Index (DLBCL-PI), and NCCN-IPI with 2031, 1803, and 1650 patients, respectively [3, 8, 12]. Five models were developed in populations larger than 500 patients [3, 8, 12]. All studies proposing models with three risk groups analyzed less than 400 patients (range 88-365) [7, 28, 32–34]. The median age in analyzed studies ranged from 57 to 70 years [8, 29, 31, 32]. Three studies reported the median age of the analyzed population over 68 years [29, 31, 32].

**Table 2:** Clinical and laboratory variables included in each model.

Models with four risk groups	Age (years)	ECOG PS	Stage	EN sites	LDH	Hgb, g/L	ALC, 10^9^/L	PLT, 10^9^/L	Albumin, g/L
IPI [3]	≤60 vs. >60	≤1 vs. >1	I/II vs. III/IV	≤1 vs. >1	≤ULN vs. >ULN				
aaIPI [3]			I/II vs. III/IV	≤1 vs. >1	≤ULN vs. >ULN				
NCCN-IPI [8]	≤40 vs. 41–60 vs. 61–75 vs. >75	≤1 vs. >1	I/II vs. III/IV	0 vs. ≥1^a^	≤ULN vs. 1–3xULN vs. >3xULN				
DLBCL-PI [12]	≤70 vs. >70	≤1 vs. >1	I/II vs. III/IV		≤ULN vs. >ULN				≤40 vs. >40
aaDLBCL-PI [12]		≤1 vs. >1		≤1 vs. >1	≤ULN vs. >ULN				≤40 vs. >40
Modified NCCN-IPI [30]	≤40 vs. 41–60 vs. 61–75 vs. >75	≤1 vs. >1	I/II vs. III/IV	0 vs. ≥1^a^	≤ULN vs. 1-3xULN vs. >3xULN				<35 vs. ≥35
KPI [31]		≤1 vs. >1		0 vs. ≥1^a^	≤ULN vs. 1-3xULN vs. >3xULN				<35 vs. ≥35
Modified 3-Factor Model [29]		≤1 vs. >1	I/II vs. III/IV				<1 vs. ≥1		
Models with three risk groups									
R-IPI [7]	≤60 vs. >60	≤1 vs. >1	I/II vs. III/IV	≤1 vs. >1	≤ULN vs. >ULN				
Matsumoto Model [33]			<III vs. ≥III			<Gr 2 vs. ≥Gr 2^b^			
ALC/R-IPI [28]	≤60 vs. >60	≤1 vs. >1	I/II vs. III/IV	≤1 vs. >1	≤ULN vs. >ULN		<0.84 vs. ≥0.84		
PA Score [32]								<100 vs. ≥100	<35 vs. ≥35
HP Index [34]						<120 vs. ≥120		<135 vs. ≥135	

*aaDLBCL-PI* age-adjusted DLBCL-PI, *aaIPI* age-adjusted IPI, *ALC* absolute lymphocyte count, *DLBCL* diffuse large B-cell lymphoma, *DLBCL-PI* DLBCL Prognostic Index, *ECOG PS* Eastern Oncology Cooperative Group performance status, *EN* Extranodal, *Hgb* hemoglobin, *HP* hemoglobin-platelet, *IPI* International Prognostic Index, *KPI* Kyoto Prognostic Index, *LDH* lactate dehydrogenase, *NCCN-IPI* National Comprehensive Cancer Network-IPI, *No* number, *PA* platelet-albumin, *PLT* platelets, *R-IPI* Revised International Prognostic Index, *ULN* upper limit of normal.

^a^Look into original publications for calculation of the high-risk localizations.

^b^Anemia grade 2: Hemoglobin <10–8.0 g/dL (Common Terminology Criteria for Adverse Events—CTCAE).

**Table 3:** Summary of variables, distributions of patients according to four risk group models, and 3/5-year overall survival in original models and models from the current study.

Model (*N* of patients)	Original study					Current study *N* = 5126		
	Model variables (points)	Risk groups (points)	% of patients	OS-years	OS (%)	*N* of patients (%)	3-year OS (%)	5-year OS (%)
IPI [3] *N* = 2031	Age >60 years (1) ECOG PS >1 (1) Stage III/IV (1) EN sites >1 (1) LDH >ULN (1)	L (0–1) LI (2) HI (3) H (4–5)	35 27 22 16	5-	73.0 51.0 43.0 26.0	1342 (26.2) 1267 (24.7) 1426 (27.8) 1091 (21.3)	92.3 80.2 69.6 49.1	85.8 74.7 62.2 43.6
aaIPI [3] >60 years *N* = 761	Stage III/IV (1) EN sites >1 (1) LDH >ULN (1)	L (0) LI (1) HI (2) H (3)	18 31 35 16	5-	56 44 37 21	1016 (19.8) 1277 (24.9) 1837 (35.8) 996 (19.4)	89.1 80.3 69.4 58.1	81.4 73.8 63.6 52.1
NCCN-IPI [8] *N* = 1650	Age ≤40 (0), 41–60 (1), 61–75 (2), >75 (3) ECOG PS >1 (1) Stage III/IV (1) EN sites ≥1 (1) LDH >1-3xULN (1), >3xULN (2)	L (0–1) LI (2) HI (3) H (4–5)	19.0 42.0 31.0 8.0	5-	96.0 82.0 64.0 33.0	414 (8.1) 1870 (36.5) 2129 (41.5) 713 (13.9)	99.2 87.6 67.8 40.9	97.1 81.8 60.1 33.4
DLBCL-PI [12] *N* = 1803	Age >70 (1) ECOG PS >1 (1) Stage III/IV (1) LDH >ULN (1) Albumin ≤40 g/L	L (0–1) LI (2) HI (3) H (4–5)	33.1 26.1 23.1 17.7	5-	87.0 69.0 53.0 37.0	1206 (23.5) 1283 (25.0) 1474 (28.8) 1163 (22,7)	93.5 83.7 70.9 46.1	89.5 76.0 64.1 38.5
aaDLBCL-PI^a^ [12] (≤70 years) *N* = 1169	ECOG PS >1 (1) EN sites >1 LDH >ULN (1) Albumin ≤40 g/L (1)	L (0) LI (1) HI (2) H (3-4)	27.2 30.8 25.3 16.7	5-	92.0 84.0 74.0 47.0	776 (15.1) 1499 (29.2) 1592 (31.1) 1259 (24.6)	91.2 85.3 71.1 52.8	85.5 77.9 64.5 47.4
Modified NCCN-IPI [30] *N* = 403	Age ≤40 (0), 41-60 (1), 61-75 (2), >75 (3) ECOG PS >1 (1) Stage III/IV (1) EN sites ≥1 (1) LDH > 1–3xULN (1), >3xULN (2) Albumin <35 g/L (1)	L (0-2) LI (3) HI (4-7) H (8-10)	24.0 19.0 49.0 8.0	5-	93.5 78.0 55.7 36.8	1049 (20.5) 935 (18.2) 2698 (52.6) 444 (8.7)	96.4 85.1 67.0 37.9	92.8 79.2 59.2 31.7
KPI [31] *N* = 323	ECOG PS >1 (1) EN sites ≥1 LDH >1–3xULN (1), >3xULN (2) Albumin <35 g/L (1)	L (0) LI (1-2)HI (3) H (4-5)	32.6 42.7 11.1 13.6	3-	96.4 84.7 63.8 33.3	1255 (24.5) 2737 (53.4) 702 (13.7) 432 (8.4)	89.4 76.2 55.1 43.7	82.3 69.9 47.9 40.3
Modified 3-factor Model [29] *N* = 274	ECOG PS >1 (1) Stage III/IV (1) ALC <1.0 x 10^9^/L (1)	Score 0 Score 1 Score 2 Score 3	32.0 34.0 25.0 9.0	3-	95.0 79.0 40.0 18.0	1249 (24.4) 2058 (40.1) 1435 (28.0) 384 (7.5)	90.2 76.8 63.0 44.9	84.5 69.7 56.8 39.4

*aaDLBCL-PI* age-adjusted DLBCL-PI, *aaIPI* age-adjusted IPI, *ALC* absolute lymphocyte count, *DLBCL* diffuse large B-cell lymphoma, *DLBCL-PI* DLBCL Prognostic Index, *ECOG PS* Eastern Oncology Cooperative Group performance status, *EN* extranodal, *IPI* International Prognostic Index, *KPI* Kyoto Prognostic Index, *LDH* lactate dehydrogenase, *NCCN-IPI* National Comprehensive Cancer Network-IPI, *N* number, *OS* overall survival, *R-IPI* Revised International Prognostic Index, *ULN* upper limit of normal.

^a^Developed for younger patients; look in the original publication.

**Table 4:** Summary of variables, distributions of patients according to three risk group models, and 3/5-year overall survival in original models and models from the current diffuse large B-cell lymphoma cohort.

Model (*N* of patients)	Original study					Current study *N* = 5126		
	Model variables (points)	Risk groups (points)	% of patients	OS-years	OS (%)	*N* of patients (%)	3-year OS (%)	5-year OS (%)
R-IPI [7] *N* = 365	Age >60 years (1) ECOG PS > 1 (1) Stage III/IV (1) EN sites >1 (1) LDH > ULN (1)	Very good (0) Good (1–2) poor (3–5)	10.0 45.0 45.0	4-	94.0 79.0 55.0	330 (6.4) 2279 (44.5) 2517 (49.1)	99.7 84.5 60.8	97.5 77.9 53.9
Matsumoto Model [33] *N* = 187	Stage ≥III (1) Anemia ≥Gr 2^a^ (1)	score 0 score 1 score 2	42.2 43.3 14.5	3-	94.6 82.0 61.4	1569 (30.6) 3015 (58.8) 543 (10.6)	86.7 70.7 54.0	80.4 64.2 48.0
ALC/R-IPI [28] *N* = 88	R-IPI poor (1) ALC < 0.84 × 10^9^/L (1)	L (0) I (1) H (2)	44.3 35.2 20.4	22 months	92.0 81.0 56.0	2227 (43.4) 2091 (40.8) 808 (15.8)	87.8 66.3 54.9	82.1 58.4 50.4
Models based on laboratory variables only								
PA Score [32] *N* = 391	Albumin <35 g/L (1) PLT < 100×10^9^/L (1)	Score 0 Score 1 Score 2	62.1 32.0 5.9	5-	81.5 48.6 20.2	3509 (68.5) 1505 (29.4) 112 (2.2)	81.4 58.2 46.8	75.1 51.6 38.9
HP Index [34] *N* = 89	PLT < 135 × 10^9^/L (1) Hgb <120 g/L (1)	Score 0 Score 1 Score 2	47.2 43.8 9.0	3-	79.0 52.0 30.0	3106 (60.6) 1813 (35.4) 207 (4.0)	83.6 66.3 51.0	75.6 56.4 42.3

*ALC* absolute lymphocyte count, *ECOG PS* Eastern Oncology Cooperative Group performance status, *EN* extranodal, *Hgb* hemoglobin, *HP* hemoglobin-platelet, *LDH* lactate dehydrogenase, *N* number, *OS* overall survival, *PA* platelet-albumin, *PLT* platelets, *R-IPI* Revised International Prognostic Index, *ULN* upper limit of normal.

^a^Anemia grade 2: Hemoglobin <10–8.0 g/dL (Common Terminology Criteria for Adverse Events—CTCAE).

### Variables included in models

The most commonly used variables in analyzed models were the IPI variables, including Ann Arbor stage (9/13), ECOG PS (9/13), LDH (9/13), age (6/13), and extranodal sites (5/13) (Table 2). In contrast to the IPI, three models stratified extranodal involvement by high-risk localizations and not the absolute number of involved sites [8, 30, 31]. Additionally, four laboratory variables (hemoglobin, platelet [PLT] count, and absolute lymphocyte count [ALC]) were used in individual models, while albumin was used in four models. Different cut-offs for age, LDH, hemoglobin, PLT, ALC, and albumin were used across different studies (Table 2).

Variables were commonly dichotomized, while age and LDH were divided into several groups in NCCN-IPI, Modified NCCN-IPI, and Kyoto Prognostic Index (KPI) [8, 30, 31].

### Model agreement

All patients were categorized into risk groups according to the prognostic models used in this analysis. Distributions of patients according to risk categories in original models and current study are provided in Tables 3 and 4.

IPI classified 26.2% of patients into low-risk groups, whereas 21.3% were in high-risk groups. aaIPI, R-IPI, and NCCN-IPI classified 19.8, 6.4%, 8.1%, respectively, in the low-risk group and 19.4%, 49.1%, and 13.9% in the high-risk group.

As presented in Suppl. Table 1, when IPI was used as the reference model and compared to other models with four-risk groups, it showed substantial agreement (weighted *κ* between 0.61-0.80) with aaIPI (weighted *κ* = 0.76), NCCN-IPI, Modified NCCN-IPI, DLBCL-PI, and age-adjusted DLBCL-PI (aaDLBCL-PI). When NCCN-IPI was used as the reference model, it showed substantial agreement with the IPI and Modified NCCN-IPI. The highest number of differently grouped patients with only fair agreement (weighted *κ* between 0.21–0.40) was observed between the NCCN-IPI vs. KPI (weighted *κ* = 0.35) and Modified-3-factor Model (weighted *κ* = 0.37). When models with three risk categories were compared with the R-IPI as the reference model, they showed poor (weighted *κ* < 0.00) to slight agreement (weighted *κ* = 0-0.20) with only ALC/R-IPI showing fair agreement with R-IPI (weighted *κ* = 0.24) (Suppl. Table 2).

### Survival

#### Overall survival (OS)

The median follow‐up of the study population was 58.2 months, and the maximum follow-up of 244.7 months. There were 2190 deaths (42.7%). The median survival of the whole study population was 135.1 months (95% CI 127.2–143.0).

Univariate analysis of parameters included in each model showed the prognostic significance of all included variables in the evaluated prognostic models (Suppl. Table 3). As several models used laboratory variables (e.g., hemoglobin, PLT, ALC, albumin) with different cut-offs of the same variable included in some models, we compared the laboratory biomarkers’ hazard ratios (HR) at different cut-offs. We then applied the cut-offs producing the highest HR in multivariate analysis. Five IPI/NCCN-IPI variables were further combined with four laboratory variables (hemoglobin<120 g/L, PLT < 100 × 10^9^/L, ALC < 0.84 × 10^9^/L, and albumin<35 g/L) in multivariate analysis. No significant correlations (collinearity) between models included in multivariate analysis were observed. In multivariate analysis with IPI parameters, only the number of extranodal sites was insignificant, while when combining five NCCN-IPI parameters with four laboratory variables, all parameters retained prognostic significance with extranodal sites marginally significant (Suppl. Table 3).

**Fig. 2:**
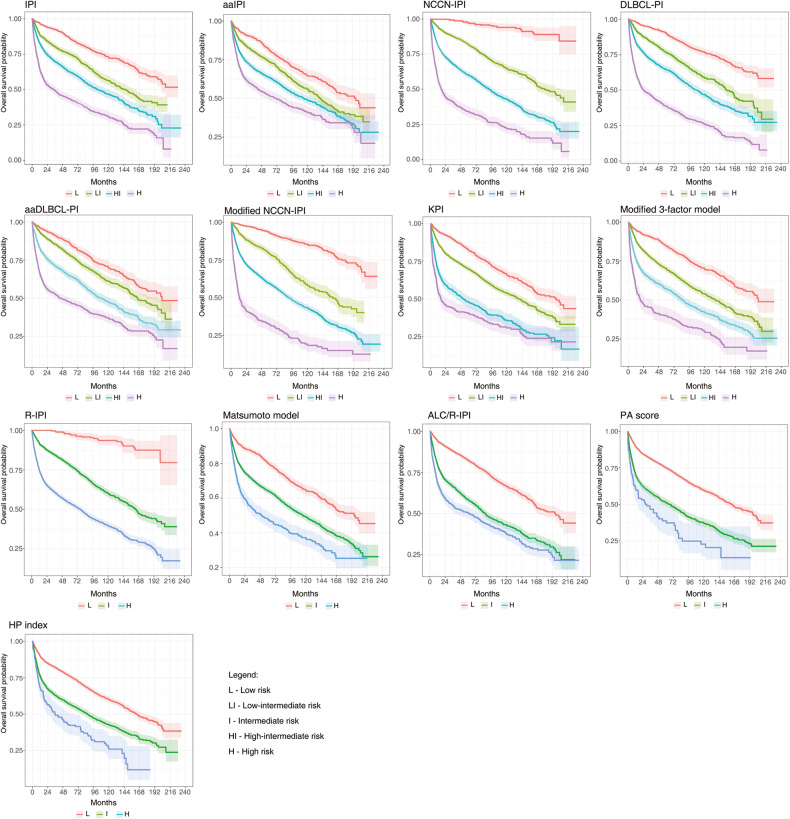
Overall survival of 13 prognostic models in diffuse large B-cell lymphoma patients (Kaplan–Meier curves). The shaded color areas around curves represent confidence intervals.

Figure 2 presents Kaplan–Meier curves for all 13 models. Moreover, we calculated 3- and 5-year OS rates for all models, as shown in Tables 3 and 4. Five-year OS estimates in the respective high-risk groups ranged from 31.7%, 33.4%, and 38.5% for Modified NCCN-IPI, NCCN-IPI, and DLBCL-PI, respectively, to 43.6% and 53.9% for IPI and R-IPI. In the respective low-risk groups, 5-year OS was 89.5%, 92.8%, and 97.1% for DLBCL-PI, Modified NCCN-IPI, and NCCN-IPI, while a lower estimate of 85.8% was registered for IPI, but not for R-IPI (97.5%).

The median PFS was 129.5 months (95% CI, 120.8–138.2 months), and the maximum PFS was 244.7 months. Suppl. Table 4 provides HRs for PFS for risk groups within each prognostic model. Moreover, measures of model fitness and discrimination are also provided in Suppl. Table 4. Kaplan–Meier and calibration curves were similar to those of OS (data not provided).

#### Model fit, discrimination, and calibration

The lowest AIC was registered for NCCN-IPI (34002), Modified NCCN-IPI (34039), and DLBCL-PI (34100). The highest AIC was registered in aaIPI (34748), along with laboratory models (Table 5). IPI and R-IPI had AIC values in the middle of the group (34340, 34380). Regarding BIC, similar results were obtained as AIC (Table 5).

**Table 5:** Summary of hazard ratios, model fit/quality measures, and discrimination measures concerning overall survival.

	HR (95% CI)	AIC	BIC	CPE	AUC	c-index	Difference in c-index
IPI [3]	Reference 1.858 (1.614; 2.138) 2.659 (2.328; 3.038) 4.758 (4.165; 5.436)	34340	34357	0.641	0.634	0.673 (0.658; 0.687)	–0.020 (–0.028; –0.012)
aaIPI [3]	Reference 1.426 (1.237; 1.643) 1.890 (1.658; 2.153) 2.583 (2.244; 2.974)	34748	34765	0.585	0.585	0.613 (0.601; 0.625)	–0.080 (–0.090; –0.069)
NCCN-IPI [8]	Reference 6.243 (4.182; 9.318) 13.470 (9.057; 20.033) 28.646 (19.156; 42.838)	34002	34019	0.670	0.657	0.693 (0.681; 0.705)	**Reference**
DLBCL-PI [12]	Reference 1.945 (1.673; 2.262) 2.963 (2.572; 3.414) 6.347 (5.521; 7.297)	34100	34117	0.660	0.661	0.700 (0.688; 0.713)	**0.007 (–0.003; 0.017)**
aaDLBCL-PI [12]	Reference 1.331 (1.138; 1.557) 2.171 (1.870; 2.521) 3.540 (3.049; 4.110)	34527	34544	0.617	0.622	0.658 (0.647; 0.668)	–0.035 (–0.046; –0.025)
Modified NCCN-IPI [30]	Reference 2.917 (2.408; 3.532) 5.363 (4.537; 6.339) 12.574 (10.362; 15.258)	34039	34056	0.664	0.651	0.684 (0.674; 0.694)	–0.009 (–0.016; –0.003)
KPI [31]	Reference 1.604 (1.427; 1.803) 3.043 (2.646; 3.500) 4.054 (3.472; 4.734)	34549	34567	0.603	0.611	0.643 (0.635; 0.651)	–0.050 (–0.060; –0.041)
Modified 3-factor Model [29]	Reference 1.827 (1.608; 2.076) 2.752 (2.417; 3.134) 4.669 (3.967; 5.495)	34538	34555	0.614	0.605	0.645 (0.627; 0.662)	–0.048 (–0.058; –0.039)
Models with three risk groups							
R-IPI [7]	Reference 6.686 (4.377; 10.210) 14.264 (9.357; 21.740)	34380	34392	0.628	0.615	0.643 (0.635; 0.652)	–0.050 (–0.058; –0.041)
Matsumoto Model [33]	Reference 1.783 (1.608; 1.978) 2.800 (2.429; 3.227)	34731	34742	0.580	0.576	0.598 (0.585; 0.612)	–0.095 (–0.107; –0.083)
ALC/R-IPI [28]	Reference 2.270 (2.059; 2.503) 2.942 (2.611; 3.314)	34537	34548	0.611	0.606	0.642 (0.633; 0.651)	–0.051 (–0.060; –0.042)
PA score [32]	Reference 2.187 (2.004; 2.387) 3.298 (2.611; 4.165)	34619	34631	0.584	0.588	0.612 (0.601; 0.622)	–0.081 (–0.091; –0.071)
HP index [34]	Reference 1.821 (1.670; 1.986) 2.815 (2.348; 3.374)	34713	34725	0.577	0.577	0.602 (0.593; 0.612)	–0.091 (–0.107; –0.074)

*aaDLBCL-PI* age-adjusted DLBCL-PI, *aaIPI* age-adjusted IPI, *AIC* Akaike Information Criterion, *ALC* absolute lymphocyte count, *AUC* area under the curve, *BIC* Bayesian Information Criterion, *c-index* concordance index, *CPE* Concordance probability estimate, *DLBCL* diffuse large B-cell lymphoma, *DLBCL-PI* DLBCL Prognostic Index, *HP* hemoglobin-platelet, *IPI* International Prognostic Index, *KPI* Kyoto Prognostic Index, N*CCN-IPI* National Comprehensive Cancer Network-IPI, *PA* platelet-albumin, *R-IPI* Revised International Prognostic Index.

The c-index is not accompanied by a p value in the statistical package used in this article. However, only the model with bold numbers does not differ from the reference model (NCCN-IPI), which can be seen from confidence intervals (CI). To be precise, *p* value for DLBCL-PI is >0.05, while it is <0.05 for all other models. We do not suggest writing *p* values in this case, as CI is more precise.

The highest CPE values were found for NCCN-IPI (0.670), Modified NCCN-IPI (0.664), and DLBCL-PI (0.660). The lowest CPE was registered for the Matsumoto model (0.580), aaIPI (0.585), and two laboratory models (0.577, 0.584) (Table 5).

**Fig. 3:**
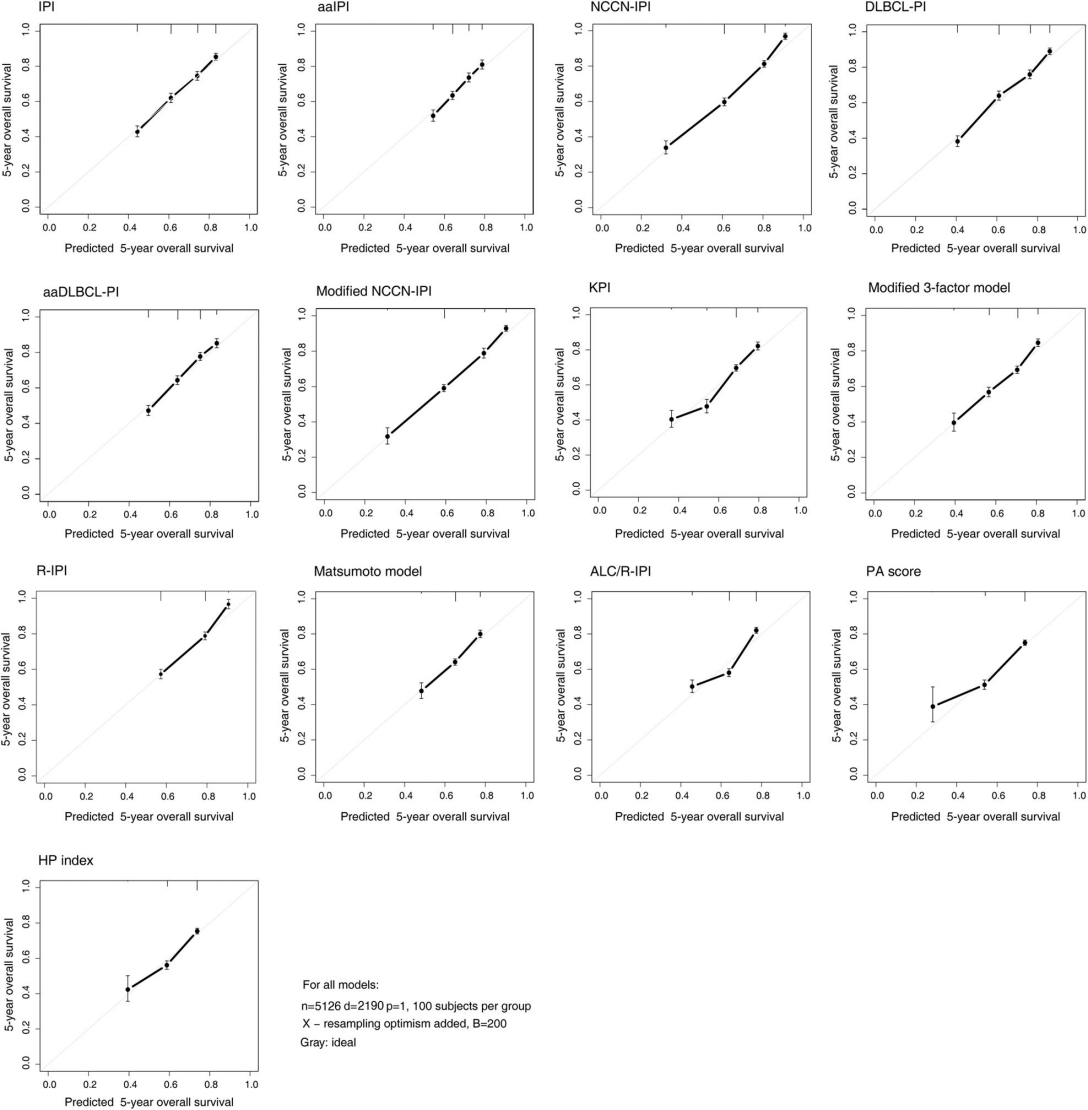
Calibration curves of 13 prognostic models in diffuse large B-cell lymphoma patients concerning overall survival.

Models that provided the highest c-index were DLBCL-PI, NCCN-IPI, and Modified NCCN-IPI, with values of 0.700, 0.693, and 0.684, respectively. When these models were compared to NCCN-IPI as the reference model, there was no statistical difference between the NCCN-IPI and DLBCL-PI. However, NCCN-IPI had statistically better discriminative ability than Modified NCCN-IPI. Additionally, NCCN-IPI performed significantly better than IPI and R-IPI, for which the c-indexes were 0.673 and 0.643, respectively. Other models with three risk groups had significantly inferior discriminative ability than NCCN-IPI with a c-index lower than R-IPI (Fig. 3).

Additionally, when AUC was calculated, DLBCL-PI, NCCN-IPI, and Modified NCCN-IPI showed the highest values (0.661, 0.657, and 0.651, respectively). In contrast, the lowest AUC values were found in three-risk models and aaIPI.

Calibration curves for 5-year survival are provided in Fig. 3. Models with the highest c-index had calibration curves close to a 45-degree line, indicating good calibration.

## Discussion

We have used a population-based lymphoma database to compare and validate 13 prognostic models for DLBCL, including two laboratory-based ones. We confirmed the prognostic value of all included models in the rituximab era but found variable discriminatory power among different models. Although previous studies have shown that the IPI has diminished value in the rituximab era, it is still widely used for risk stratification in general practice and clinical trials. So far, only a few models have been previously validated in different populations (e.g., R-IPI, NCCN-IPI) [2, 15, 35]. Our study shows that although an ideal clinical model is still needed, NCCN-IPI and DLBCL-PI provided better discriminative ability than other analyzed models in particular models with three-risk groups and aaIPI [8, 12].

The predictive capacity of the IPI has been universally accepted due to the use of robust and easily accessible markers. Age was frequently included in analyzed models, as its relevance with different cut-offs has been confirmed in DLBCL in numerous studies [8, 12, 30, 36]. In our analysis, age was incorporated in all three models providing the highest discrimination, with two incorporating fractionalized age groups (NCCN-IPI, Modified NCCN-IPI) [8, 30]. Accordingly, Biccler et al. reported a significant loss in predictive performance when dichotomizing age, although this was not the case with the dichotomization of other IPI variables [11]. Age is probably one of the most influential survival predictors as it captures some patient characteristics not directly included in models. Although in elderly patients, lymphoma is not substantially less responsive to treatment than in younger patients, poor outcome in elderly patients results mainly from their decreased ability to tolerate treatment [37]. Moreover, polypharmacy, changes in drug metabolism with age, comorbidities, and impaired bone marrow function increase the risk of treatment-related toxicity [37]. Therefore, deaths from other causes, specifically in the elderly, represent a significant competing risk [11].

In our study, all five IPI/NCCN-IPI variables were significant in univariate analysis but not multivariate analysis. When IPI/NCCN-IPI variables were combined with the five laboratory variables included in other models, only extranodal sites lost prognostic significance. As the prognostic impact of the extranodal scoring system included in the IPI has been questioned in the rituximab era, numerous studies have proposed that the particular (number of) high-risk sites are more impactful than the number of involved sites according to the IPI [8, 31, 38, 39]. This distinction has been considered in three models we evaluated [8, 30, 31]. Incorporating positron emission tomography measures (i.e., baseline metabolic tumor volume [MTV]) instead of extranodal sites could further improve the value of future models for DLBCL [40–44]. However, standardization across studies evaluating these measures is currently lacking [40–43].

Several studies have previously demonstrated the prognostic value of albumin [12, 45, 46]. Low albumin levels are associated with poor general health, inflammation, and low body mass index (BMI) and are generally related to poor survival in patients independent of lymphoma [45]. Of the four models incorporating albumin levels, only DLBCL-PI and Modified NCCN-IPI showed superior performance measures than the other models [12, 30]. As DLBCL-PI was developed from a Danish population, overlapping with the current study is likely and could potentially lead to an overestimation of the discriminatory ability of DLBCL-PI [12]. Models incorporating albumin are challenged primarily due to missing values in retrospective cohorts, variable cut-off levels, and correlation with other conditions (e.g., previous/concomitant cancers, renal failure, low BMI, inflammatory diseases).

Laboratory markers, such as blood counts, have been investigated as potential surrogate markers of the host’s adaptive immunity and immune microenvironment [28, 47]. ALC was incorporated in two models, but the discriminative value of these models was below that of the IPI in our analysis [28, 29]. Moreover, pretreatment hemoglobin concentration was previously associated with outcomes in DLBCL patients treated with anthracycline-containing chemotherapy [48]. Although hemoglobin, PLT levels, and ALC showed prognostic significance in multivariate analysis, models incorporating these parameters had poor discrimination and calibration in the current analysis [32–34].

When we examined the prognostic model’s ability to discriminate between risk groups, all models were successful but with variable performance. Most models failed to identify populations with very poor outcomes and less than 50% long-term survival. A 5-year survival of 35% or less in high-risk groups was identified by Modified NCCN-IPI (31.7%) and NCCN-IPI (33.4%) [8, 30]. Only NCCN-IPI could identify low-risk patients with excellent prognoses with 5-year OS over 95% (97.1%) [8]. In contrast, IPI, aaIPI, and models with three-risk groups could not identify a 5-year OS of less than 40% in high-risk patients, and they also failed to identify a population with a favorable prognosis in the low-risk group with 5-year OS over 90% [3, 7, 28, 32–34]. Only R-IPI could identify low-risk patients with a 5-year OS of 97.5%, comparable to NCCN-IPI [7, 8]. Ruppert et al., using data from 7 clinical trials (2124 patients), reported 5-year OS for IPI, R-IPI, and NCCN-IPI ranging from 54% to 88%, 61% to 93%, and 49% to 92%, in the high and low-risk groups, respectively [2]. The inferior discriminatory ability of NCCN-IPI in the study of Ruppert et al. compared to the current study is likely due to the utilization of younger patients in the former study (median 63 vs. 68 years) [2]. Moreover, the authors included a population from clinical trials, while NCCN-IPI was developed from an unselected population of newly diagnosed DLBCL patients, which was also the case in our study [8].

We found superior discriminatory ability of NCCN-IPI, DLBCL-PI, and Modified NCCN-IPI compared to all other models [8, 12, 30]. Regarding the model’s discriminative ability, only DLBCL-PI showed a c-value comparable, but not superior, to the NCCN-IPI [8, 12]. The lowest discriminatory ability was observed in models with three risk groups without age as a model component, including aaIPI and aaDLBCL-PI. Moreover, when comparing the two laboratory models to the clinical models, including NCCN-IPI (c-index 0.693), they showed significantly inferior discriminatory ability (c-index ranging from 0.606 to 0.612), indicating that the established laboratory variables cannot be used alone to prognosticate DLBCL. Reasons for poor discrimination could be that these models stratified patients into only three risk groups. Moreover, clinical variables seem to add significantly to the performance of each model, probably due to the robustness of clinical variables such as age and ECOG PS. Similarly, the best fit evaluated by AIC and BIC was also registered for three previously mentioned models [8, 12, 30]. Additionally, calibration curves were in accordance with the discriminative power of different models showing poor calibration among three-risk models.

Numerous biomarkers have already been incorporated in some predictive models for DLBCL (MTV, cell of origin, genetic markers) [13, 40, 42]. Moreover, the prognostic potential of whole-genome sequencing, circulating tumor DNA (ctDNA), along with sociodemographic, clinical, laboratory, molecular, and genetic markers, are under investigation to facilitate prognostication and treatment decision-making [1]. The need for a comprehensive prognostic model for DLBCL patients has been recognized, particularly in the evolving treatment possibilities and improvements in diagnostics [1, 4]. Identifying high-risk patients who could benefit from early treatment with CAR-T, bispecifics, and other novel therapies is necessary. Studies aiming to develop more accurate models for outcome predictions should focus on using different model formulations with more predictors (including MTV, ctDNA, genetic markers), different flexible modeling approaches (e.g., spline models, random survival forests), optimal end-point (cause-specific survival, disease-free survival), and timing of model calculation (e.g., at diagnosis, interim evaluation) [11, 17, 49]. However, until new biomarkers are integrated into validated prognostic models, NCCN-IPI and the IPI should be reported for patients treated in clinical trials to allow optimal comparison of outcomes with previous studies [2]. Despite not affecting treatment decisions, NCCN-IPI can provide insight into patients’ long-term survival.

Although this is one of the most extensive validation studies of different clinical prognostic models in DLBCL based on easily obtained clinical features, several limitations should be addressed. The retrospective nature and usage of register-based data come with an inevitable bias. The risk of incorrect disease classification is possible as some cases (e.g., primary mediastinal B-cell lymphoma, primary effusion lymphoma, leg-type DLBCL) could have been registered as DLBCL. As these subtypes are rare compared to DLBCL, potential influence is likely insignificant. Moreover, we did not analyze models incorporating cell of origin, MYC/BCL2/6 re-arrangements, comorbidities, and results of interim response analysis. Models incorporating variables not reported regularly in the database and those with significant missing values were not analyzed. Additionally, overall survival, and death as a primary event, irrespective of cause, was the primary point of this study. However, an issue in survival analysis is competing risks of non-fatal outcomes and mortality due to non-lymphoma causes, which can lead to an overestimation of absolute risk [50]. Nevertheless, the study’s main strengths are the large number of patients and the direct comparison of risk indices calculated on the same individual patient from a real-world dataset.

## Conclusion

This large retrospective register-based study analyzing 13 prognostic models for DLBCL showed superior model quality and discriminatory ability of NCCN-IPI and DLBCL-PI compared to other models. Moreover, aaIPI performed poorly and should be cautiously used. Laboratory-based models with currently available markers should be critically analyzed due to the significant additive effects of clinical characteristics on prognosis. Current analysis favors NCCN-IPI, and this model is suggested as the reference model along with the IPI when developing models for DLBCL. Future developments and a better understanding of disease pathology will hopefully allow the use of the extensive data to create models based on prognostically important clinical, molecular, and genetic factors to facilitate treatment decision-making.

### Supplementary information

Suppl. Figure 1. Distribution of missing variables among 6075 potential DLBCL candidates.

Suppl. Table 1. Agreement between the risk groups of IPI, NCCN-IPI, and other clinical models with four-risk groups evaluated using weighted Cohen κ

Suppl. Table 2. Agreement between the risk groups of R-IPI and models with three-risk groups evaluated using weighted Cohen κ

Suppl. Table 3. Univariate and multivariate analysis of IPI/NCCN-IPI variables and laboratory variables

Suppl. Table 4. Summary of hazard ratios, model fit/quality measures, and discrimination measures concerning progression-free survival

## Data Availability

The datasets generated during and/or analyzed during the current study are available from the corresponding author upon reasonable request.
