# Supplementary material for: Prognostic indices in diffuse large B-cell lymphoma: a population-based comparison and validation study of multiple models
Source: Blood Cancer J. 2023 Oct 13;13(1):157. doi: 10.1038/s41408-023-00930-7 (PMC10575851; doi:10.1038/s41408-023-00930-7)
Supplement: Supplementary file 1 — Suppl. Figure 1. Distribution of missing variables among 6075 potential DLBCL candidates. [file 41408_2023_930_MOESM1_ESM.docx]

**Suppl. Figure 1**. Distribution of missing variables among 6075 potential DLBCL candidates.

^Legend: ALC – absolute lymphocyte count; DLBCL – Diffuse large B-cell lymphoma; ECOG PS – Eastern Oncology Cooperative Group performance status; EN – Extranodal; LDH – lactate dehydrogenase; N – number; β2M – beta-2 microglobulin^
