# Supplementary material for: Prognostic indices in diffuse large B-cell lymphoma: a population-based comparison and validation study of multiple models
Source: Blood Cancer J. 2023 Oct 13;13(1):157. doi: 10.1038/s41408-023-00930-7 (PMC10575851; doi:10.1038/s41408-023-00930-7)
Supplement: Supplementary file 2 — Suppl. Table 1. Agreement between the risk groups of IPI, NCCN-IPI, and other clinical models with four-risk groups evaluated using weighted Cohen κ [file 41408_2023_930_MOESM2_ESM.docx]

**Suppl. Table 1**. Agreement between the risk groups of IPI, NCCN-IPI, and other clinical models with four-risk groups evaluated using weighted Cohen κ

|  | IPI  (N=5126) | | | | NCCN-IPI  (N=5126) | | | |
| --- | --- | --- | --- | --- | --- | --- | --- | --- |
| Prognostic  models | N misclassified patients (%) | N misclassified patients  within IPI risk groups (%) | N of equally classified patients within IPI (%) | weighted κ* | N misclassified patients (%) | N misclassified patients within NCCN-IPI risk groups (%) | N equally classified patients within NCCN-IPI (%) | weighted κ |
| IPI ^3^ | / | / | / | / | 2015 (39.3) | 5 (1.2)  1029 (55.0)  897 (42.1)  84 (11.8) | 409 (98.8)  841 (45.0)  1232 (57.9)  629 (88.2) | 0.638  (0.625; 0.650) |
| aaIPI ^3^ | 1465 (28.5) | 372 (27.7)  463 (36.5)  318 (22.3)  312 (28.6) | 970 (72.3)  804 (63.5)  1108 (77.7)  779 (71.4 | 0.758  (0.747; 0.769) | 2420 (47.2) | 90 (21.7)  1093 (58.4)  956 (44.9)  281 (39.4) | 324 (78.3)  777 (41.6)  1173 (55.1)  432 (60.6) | 0.513  (0.498; 0.529) |
| NCCN-IPI ^8^ | 2015 (39.3) | 933 (69.5)  426 (33.6)  194 (13.6)  462 (42.3) | 409 (30.5)  841 (66.4)  1232 (86.4)  629 (57.7) | 0.638  (0.625; 0.650) | / | / | / | / |
| DLBCL-PI ^12^ | 2193 (42.8) | 417 (31.1)  751 (59.3)  725 (50.8)  300 (27.5) | 925 (68.9)  516 (40.7)  701 (49.2)  791 (72.5) | 0.634  (0.621; 0.648) | 2251 (43.9) | 40 (9.7)  1091 (58.3)  1037 (48.7)  83 (11.6) | 374 (90.3)  779 (41.7)  1092 (51.3)  630 (88.4) | 0.582  (0.568; 0.596) |
| aaDLBCL-PI ^12^ | 2097 (40.9) | 750 (55.9)  688 (54.3)  546 (38.3)  113 (10.4) | 592 (44.1)  579 (45.7)  880 (61.7)  978 (89.6) | 0.636  (0.622; 0.650) | 2390 (46.6) | 180 (43.5)  975 (52.1)  1123 (52.7)  112 (15.7) | 234 (56.5)  895 (47.9)  1006 (47.3)  601 (84.3) | 0.507  (0.491; 0.523) |
| Modified NCCN-IPI ^30^ | 1864 (36.4) | 382 (28.5)  666 (52.6)  124 (8.7)  692 (63.4) | 960 (71.5)  601 (47.4)  1302 (91.3)  399 (36.6) | 0.654  (0.640; 0.668) | 1242 (24.2) | 19 (4.5)  954 (51.0)  0 (0)  269 (37.7) | 395 (95.4)  916 (49.0)  2129 (100.0)  444 (62.3) | 0.731  (0.731; 0.755) |
| Modified 3-factor Model ^29^ | 2493 (48.6) | 346 (25.8)  542 (42.8)  832 (58.3)  773 (70.9) | 996 (74.2)  725 (57.2)  594 (41.7)  318 (29.1) | 0.517  (0.501; 0.533) | 2962 (57.8) | 73 (17.6)  1104 (59.0)  1312 (61.6)  473 (66.3) | 341 (82.4)  766 (41.0)  817 (38.4)  240 (33.7) | 0.375  (0.358; 0.391) |
| KPI ^31^ | 2729 (53.2) | 424 (31.6)  383 (30.2)  1196 (83.9)  726 (66.5) | 918 (68.4)  884 (69.8)  230 (16.1)  365 (33.5) | 0.473  (0.458; 0.489) | 3078 (60.0) | 102 (24.6)  865 (46.3)  1722 (80.9)  389 (54.6) | 312 (75.4)  1005 (53.7)  407 (19.1)  324 (45.4) | 0.355  (0.339; 0.371) |

^aaDLBCL-PI – age-adjusted DLBCL-PI; aaIPI – age-adjusted IPI; DLBCL – Diffuse large B-cell lymphoma; DLBCL-PI – DLBCL Prognostic Index; HP - hemoglobin-platelet; IPI – International Prognostic Index; KPI – Kyoto Prognostic Index; N – number; NCCN-IPI – National Comprehensive Cancer Network-IPI; PA – platelet-albumin; R-IPI – Revised International Prognostic Index^

^Cohen κ: <0.00 poor; 0-0.2 slight; 0.21-0.40 fair; 0.41-0.60 moderate; 0.61-0.80 substantial; 0.81-1.00 almost perfect agreement^
