# Supplementary material for: Prognostic indices in diffuse large B-cell lymphoma: a population-based comparison and validation study of multiple models
Source: Blood Cancer J. 2023 Oct 13;13(1):157. doi: 10.1038/s41408-023-00930-7 (PMC10575851; doi:10.1038/s41408-023-00930-7)
Supplement: Supplementary file 3 — Suppl. Table 2. Agreement between the risk groups of R-IPI and models with three-risk groups evaluated using weighted Cohen κ [file 41408_2023_930_MOESM3_ESM.docx]

**Suppl. Table 2**. Agreement between the risk groups of R-IPI and models with three-risk groups evaluated using weighted Cohen κ

|  | N of misclassified patients (%) | N of misclassified patients  within R-IPI risk groups (%) | N of equally classified patients within R-IPI (%) | weighted κ |
| --- | --- | --- | --- | --- |
| R-IPI ^7^  (N=5126) |  | / | / | / |
| Matsumoto Model ^33^ | 3338 (65.1) | 5 (1.5)  1276 (56.0)  2057 (81.7) | 325 (98.5)  1003 (44.0)  460 (18.3) | 0.198 (0.183; 0.212) |
| ALC/R-IPI ^28^ | 3628 (70.8) | 11 (3.3)  1908 (83.7)  1709 (67.9) | 319 (96.7)  371 (16.3)  808 (32.1) | 0.244 (0.232; 0.257) |
| PA score ^32^ | 4304 (84.0) | 10 (3.0)  1867 (81.9)  2427 (96.4) | 320 (97.0)  412 (18.1)  90 (3.6) | 0.044 (0.038; 0.049) |
| HP index ^34^ | 4083 (76.6) | 30 (9.1)  1696 (74.4)  2357 (93.6) | 300 (90.9)  583 (25.6)  160 (6.4) | 0.057 (0.049; 0.065) |

^ALC – absolute lymphocyte count; N – number; HP - hemoglobin-platelet; PA – platelet-albumin; R-IPI – Revised International Prognostic Index^

^Cohen κ: <0.00 poor; 0-0.2 slight; 0.21-0.40 fair; 0.41-0.60 moderate; 0.61-0.80 substantial; 0.81-1.00 almost perfect agreement^
