# Supplementary material for: Prognostic indices in diffuse large B-cell lymphoma: a population-based comparison and validation study of multiple models
Source: Blood Cancer J. 2023 Oct 13;13(1):157. doi: 10.1038/s41408-023-00930-7 (PMC10575851; doi:10.1038/s41408-023-00930-7)
Supplement: Supplementary file 4 — Suppl. Table 3. Univariate and multivariate analysis of IPI/NCCN-IPI variables and laboratory variables [file 41408_2023_930_MOESM4_ESM.docx]

**Suppl. Table 3**. Univariate and multivariate analysis of IPI/NCCN-IPI variables and laboratory variables

|  | univariate analysis |  | multivariate analysis with IPI variables | | multivariate analysis with NCCN-IPI variables | |
| --- | --- | --- | --- | --- | --- | --- |
| Variables | HR, 95 %CI | p-value | HR, 95% CI | p-value | HR, 95% CI | p-value |
| Age ≤60 vs. >60 years | 3.779 (3.341; 4.275) | <0.001 | 3.551 (3.136; 4.021) | <0.001 |  |  |
| Age ≤40  41-60  61-75  >75 | Reference  4.558 (2.794; 7.439)  11.000 (6.810; 17.768)  24.083 (14.887; 38.960) | <0.001  <0.001  <0.001  <0.001 |  |  | Reference  4.284 (2.624; 6.993)  9.954 (6.157; 16.090)  21.734 (13.422; 35.193) | <0.001  <0.001  <0.001  <0.001 |
| Stage I/II vs. III/IV | 1.832 (1.660; 2.021) | <0.001 | 1.210 (1.083; 1.352) | 0.001 | 1.225 (1.097; 1.368) | <0.001 |
| ECOG PS 0-1 vs. ≥2 | 2.904 (2.641; 3.192) | <0.001 | 1.935 (1.745; 2.146) | <0.001 | 1.790 (1.616; 1.984) | <0.001 |
| No EN sites (IPI) 1 vs. >1 | 1.448 (1.326; 1.581) | <0.001 | 1.065 (0.969; 1.171) | **0.190** |  |  |
| EN sites (NCCN-IPI) <1 vs. ≥1 | 1.503 (1.378; 1.639) | <0.001 |  |  | 1.099 (1.001; 1.207) | 0.048 |
| EN KPI <1 vs. ≥1 | 1.482 (1.362; 1.612) | <0.001 |  |  |  |  |
| LDH IPI normal vs. >ULN | 1.534 (1.407; 1.672) | <0.001 | 1.213 (1.105; 1.331) | <0.001 |  |  |
| LDH ≤ ULN  >1-3xULN  >3x ULN | Reference  1.417 (1.294; 1.551)  2.214 (1.937; 2.531) | <0.001  <0.001  <0.001 |  |  | Reference  1.286 (1.168; 1.416)  1.466 (1.267; 1.696) | <0.001  <0.001  <0.001 |
| Hgb grade 2* yes vs. no | 1.838 (1.640; 2.060) | <0.001 |  |  |  |  |
| Hgb <120x10^9^/L yes vs. no | 1.840 (1.692; 2.001) | <0.001 | 1.182 (1.075; 1.299) | 0.001 | 1.153 (1.049; 1.267) | 0.003 |
| PLT <100x10^9^/L yes vs. no | 2.245 (1.864; 2.704) | <0.001 | 1.543 (1.275; 1.866) | <0.001 | 1.557 (1.286; 1.887) | <0.001 |
| PLT <135x10^9^/L yes vs. no | 2.004 (1.728; 2.324) | <0.001 |  |  |  |  |
| ALC <1.0x10^9^/L yes vs. no | 1.531 (1.405; 1.667) | <0.001 |  |  |  |  |
| ALC <0.84x10^9^/L yes vs. no | 1.548 (1.411; 1.699) | <0.001 | 1.171 (1.063; 1.291) | 0.001 | 1.155 (1.048; 1.273) | 0.004 |
| Albumin <35g/L yes vs. no | 2.199 (2.019; 2.397) | <0.001 | 1.397 (1.267; 1.541) | <0.001 | 1.362 (1.235; 1.502) | <0.001 |
| Albumin ≤40g/L yes vs. no | 2.034 (1.845; 2.242) | <0.001 |  |  |  |  |

^ALC – Absolute lymphocyte count; CI – Confidence interval; ECOG PS – Eastern Oncology Cooperative Group performance status; EN – Extranodal; Hgb – hemoglobin; HR – hazard ratio; IPI – International Prognostic Index; KPI – Kyoto Prognostic Index LDH – lactate dehydrogenase; NCCN-IPI – National Comprehensive Cancer Network-IPI; PLT – platelets; ULN – upper limit of normal^

^**Anemia grade 2: Hemoglobin <10 – 8.0 g/dL (Common Terminology Criteria for Adverse Events – CTCAE)^
