# Supplementary material for: Prognostic indices in diffuse large B-cell lymphoma: a population-based comparison and validation study of multiple models
Source: Blood Cancer J. 2023 Oct 13;13(1):157. doi: 10.1038/s41408-023-00930-7 (PMC10575851; doi:10.1038/s41408-023-00930-7)
Supplement: Supplementary file 5 — Suppl. Table 4. Summary of hazard ratios, model fit/quality measures, and discrimination measures concerning progression-free survival [file 41408_2023_930_MOESM5_ESM.docx]

**Suppl. Table 4**. Summary of hazard ratios, model fit/quality measures, and discrimination measures concerning progression-free survival

| Models | HR (95% CI) | AIC | BIC | CPE | AUC | c-index |  |
| --- | --- | --- | --- | --- | --- | --- | --- |
| IPI ^3^ | Reference  1.897 (1.648; 2.183)  2.694 (2.358; 3.077)  4.727 (4.138; 5.400) | 34387 | 34404 | 0.640 | 0.626 | 0.666 (0.657; 0.676) | -0.017 (-0.024; -0.010) |
| aaIPI ^3^ | Reference  1.467 (1.272; 1.691)  1.963 (1.723; 2.237)  2.627 (2.282; 3.024) | 34780 | 34797 | 0.586 | 0.577 | 0.612 (0.598; 0.626) | -0.072 (-0.081; -0.062) |
| NCCN-IPI ^8^ | Reference  6.322 (4.235; 9.437)  13.483 (9.066; 20.053)  28.116 (18.802; 42.044) | 34063 | 34080 | 0.668 | 0.646 | 0.683 (0.671; 0.696) | **reference** |
| DLBCL-PI ^12^ | Reference  1.950 (1.677; 2.268)  2.959 (2.568; 3.409)  6.215 (5.406; 7.145) | 34161 | 34178 | 0.659 | 0.652 | 0.690 (0.676; 0.703) | **0.006 (0.000; 0.013**) |
| aaDLBCL-PI ^12^ | Reference  1.325 (1.132; 1.550)  2.159 (1.860; 2.506)  3.513 (3.025; 4.078) | 34572 | 34589 | 0.617 | 0.612 | 0.648 (0.632; 0.664) | -0.036 (-0.046; -0.025) |
| Modified NCCN-IPI ^30^ | Reference  2.978 (2.459; 3.606)  5.349 (4.525; 6.323)  12.281 (10.121; 14.902) | 34096 | 34113 | 0.662 | 0.641 | 0.677 (0.670; 0.684) | **-0.006 (-0.013; 0.000)** |
| KPI ^31^ | Reference  1.627 (1.448; 1.829)  3.059 (2.660; 3.518)  4.117 (3.525; 4.807 | 34586 | 34603 | 0.604 | 0.603 | 0.638 (0.628; 0.648) | -0.046 (-0.052; -0.039) |
| Modified 3-factor Model ^29^ | Reference  1.890 (1.663; 2.148)  2.828 (2.484; 3.221)  4.841 (4.112; 5.698) | 34561 | 34578 | 0.616 | 0.603 | 0.642 (0.630; 0.655) | -0.041 (-0.049; -0.033) |
| Models with three-risk groups | | | | |  |  |  |
| R-IPI ^7^ | Reference  6.693 (4.382; 10.230)  14.228 (9.334; 21.690) | 34424 | 34436 | 0.627 | 0.608 | 0.638 (0.629; 0.647) | -0.046 (-0.052; -0.039) |
| Matsumoto Model ^33^ | Reference  1.831 (1.651; 2.030)  2.862 (2.483; 3.299) | 34758 | 34770 | 0.582 | 0.572 | 0.599 (0.584; 0.613) | -0.085 (-0.097; -0.072) |
| ALC/R-IPI ^28^ | Reference  2.291 (2.079; 2.526)  2.931 (2.602; 3.302) | 34575 | 34586 | 0.611 | 0.601 | 0.635 (0.622; 0.649) | -0.048 (-0.058; -0.039) |
| PA score ^32^ | Reference  2.167 (1.986; 2.365)  3.299 (2.613; 4.167) | 34667 | 34678 | 0.583 | 0.578 | 0.607 (0.600; 0.615) | -0.076 (-0.086; -0.066) |
| HP index ^34^ | Reference  1.840 (1.687; 2.006)  2.825 (2.356; 3.386) | 34749 | 34760 | 0.578 | 0.574 | 0.597 (0.586; 0.608) | -0.086 (-0.098; -0.075) |

^aaDLBCL-PI – age-adjusted DLBCL-PI; aaIPI – age-adjusted IPI; AIC – Akaike Information Criterion; ALC – absolute lymphocyte count; AUC – Area under the curve; BIC – Bayesian Information Criterion; c-index – concordance index; CPE – Concordance probability estimate; DLBCL – Diffuse large B-cell lymphoma; DLBCL-PI – DLBCL Prognostic Index; KPI – Kyoto Prognostic Index; HP - hemoglobin-platelet; IPI – International Prognostic Index; NCCN-IPI – National Comprehensive Cancer Network-IPI; PA – platelet-albumin; R-IPI – Revised International Prognostic Index^
